# Insecticide resistance in the bed bug comes with a cost

**DOI:** 10.1038/srep10807

**Published:** 2015-06-03

**Authors:** Jennifer R. Gordon, Michael F. Potter, Kenneth F. Haynes

**Affiliations:** 1 Department of Entomology, University of Kentucky, Lexington, KY 40546-0091, USA

## Abstract

Adaptation to new environmental stress is often associated with an alteration of one or more life history parameters. Insecticide resistant populations of insects often have reduced fitness relative to susceptible populations in insecticide free environments. Our previous work showed that three populations of bed bugs, *Cimex lectularius* L., evolved significantly increased levels of resistance to one product containing both β-cyfluthrin and imidacloprid insecticides with only one generation of selection, which gave us an opportunity to explore potential tradeoffs between life history parameters and resistance using susceptible and resistant strains of the same populations. Life history tables were compiled by collecting weekly data on mortality and fecundity of bugs from each strain and treatment throughout their lives. Selection led to a male-biased sex ratio, shortened oviposition period, and decreased life-time reproductive rate. Generation time was shortened by selection, a change that represents a benefit rather than a cost. Using these life history characteristics we calculated that there would be a 90% return to pre-selection levels of susceptibility within 2- 6.5 generations depending on strain. The significant fitness costs associated with resistance suggest that insecticide rotation or utilization of non-insecticidal control tactics could be part of an effective resistance management strategy.

Adaptation to a new environmental stress is often associated with an alteration of one or more life history parameters^1^,^2^. Ultimately, these tradeoffs may be the result of a physiological constraint, such as shunting resources into survival in the new environment; resources that could then not be used for egg production or rapid development. Genetic correlations between life history traits and genes that influence resistance may explain the intergenerational response. In the case of insecticide resistance, increased production of enzymes leading to insecticide detoxification or increased production of cuticular components that reduce penetration of the toxicant may have correlated effects on life history characters. Among various species, reduced longevity, delayed maturation and decreased egg production have been observed to accompany insecticide resistance^3^,^4^,^5^,^6^,^7^,^8^. The net result of the adaptations to insecticide exposure is enhanced fitness in insecticide-treated habitats, but decreased fitness in insecticide-free areas.

Such tradeoffs open the possibility of insecticide resistance management by rotation between compounds with not only different modes of action but also different modes of detoxification (and hence different physiological costs) or to non-insecticidal control tactics. When there are negative life history tradeoffs associated with resistance in insecticide-free areas, populations should revert toward susceptiblity^9^,^10^,^11^. If no costs existed in resistant individuals relative to susceptible, insecticide resistance management via rotation would be ineffective^12^.

In the past ten to fifteen years there has been a resurgence of pyrethroid resistant populations of *Cimex lectularius* L., the bed bug^13^,^14^,^15^,^16^. Pyrethroid resistance has been found in North America, Australia, Asia and Europe and is widespread throughout the United States and presumably elsewhere^17^,^18^,^19^,^20^. In the United States, resistance to pyrethroid-only insecticides has prompted a shift by urban pest management professionals to commercial insecticide products containing both a pyrethroid and a neonicotinoid^21^,^22^. These two classes of insecticides act at different target sites on the insect neuron^23^,^24^.

Our previous work investigating the evolutionary response of multiple populations of bed bugs to one of these combination products, Temprid SC^®^ (β-cyfluthrin and imidacloprid), showed that resistance began to evolve in one generation in the laboratory^22^. This rapid evolution under laboratory conditions gave us an opportunity to explore the hypothesis that life history costs would be associated with decreased susceptibility to the combination insecticide in an environment no longer containing the insecticide.

## Results

**Figure 1:**
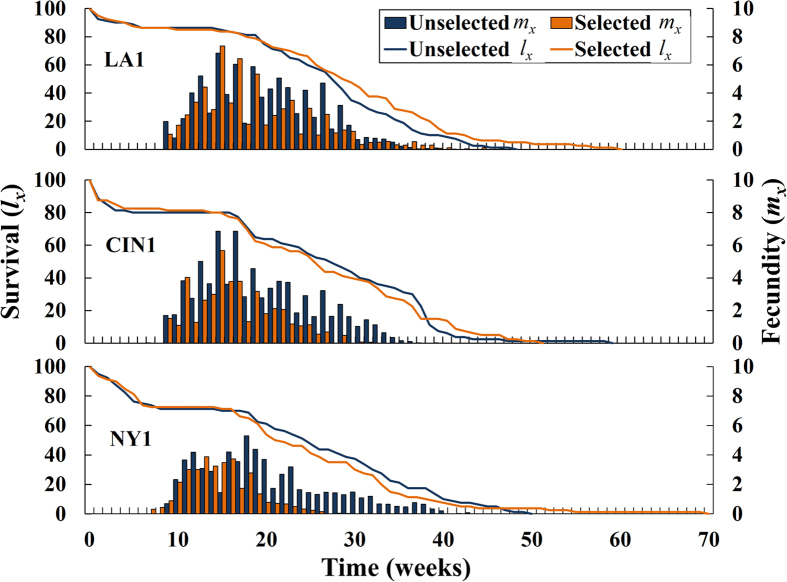
Survival (*l*_*x*_; left axis and curve) and fecundity (*m*_*x*_; right axis and histogram) over time for unselected and selected strains. Survival analysis found no significant differences in longevity due to selection with insecticide or strain (see Supplemental Table S3 for full descriptive statistics from survival analysis). Adult survival reached 50% at 31.1 (±0.94), 31.6 (±2.60) and 34.0 (±3.30) weeks for the unselected LA1, CIN1 and NY1 strains, respectively, and at 32.5 (±1.58), 30.6 (±2.34) and 28.6 (±2.77) weeks for the paired selected strains. The average time from the egg to adult molt was 10.8 (±0.63), 10.0 (±0.70), 10.3 (±0.25) weeks for the unselected LA1, CIN1 and NY1 strains, respectively, and 10.5 (±0.87), 9.3 (±0.63), 10.3 (±0.85) weeks for the paired selected strains. The average number of eggs laid during an individual female’s lifetime was 165.8 (±17.7), 154.0 (±24.9), 126.8 (±11.6) for the unselected LA1, CIN1 and NY1 strains, respectively, and 134.2 (±11.5), 84.9 (±9.7), 65.7 (±13.9) for the paired selected strains.

**Figure 2:**
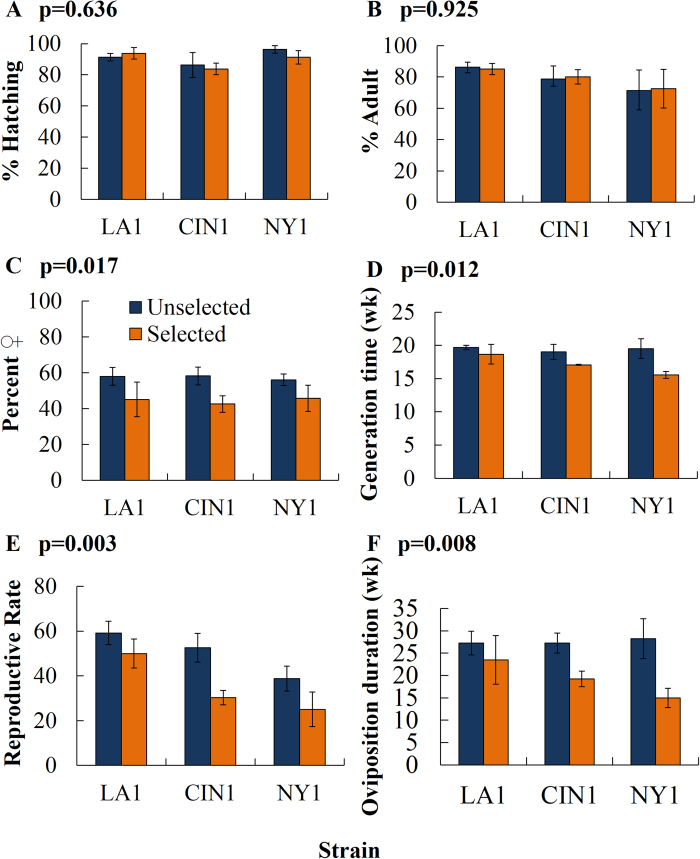
Life history parameters investigated. Selection did not affect the per cent of eggs that hatched or the per cent of eggs that reached adulthood. However, selection significantly decreased the proportion of individuals that were female, generation time, reproductive rate and oviposition duration. All results from the analysis of variance are presented in Supplemental Table S1, and p-values depicted in the figure represents the effect of selection on the specific parameter investigated.

Mortality levels caused by the selected exposure times to label-rate Temprid SC in the second filial generation of the unselected line were very similar to the parental lines as would be expected in the absence of genetic drift (Table 1). Mortality in selected lines of LA1, CIN1 and NY1 were reduced by the selection that had occurred two generations earlier. This historical selection affected some, but not all, measured life history parameters (Figs. 1 and 2, see Supplemental Table S1 for analysis of variance results). Longevity, per cent hatch and per cent reaching the adult stage were not affected by the history of selection (Figs. 1, 2A,B). Unexpectedly, the sex ratio at maturity was biased towards females in unselected lines and biased towards males in selected lines (Fig. 2C). The generation time (*G*) was significantly less in all populations with a history of insecticide exposure compared to those not selected (Fig. 2D). Generation times were significantly reduced by selection by 5.1, 10.3 and 20.2% in LA1, CIN1, and NY1, respectively. Reproductive rates (*R*_*o*_) were significantly lower in selected lines compared to their unselected counterparts (Fig. 2E). Reproductive rates were significantly reduced by 15.5, 42.5 and 35.3% in LA1, CIN1 and NY1, respectively. Additionally, the oviposition duration from the first egg laid to the last egg laid was significantly shorter for selected compared to unselected groups (Fig. 2F). Oviposition durations were reduced by 13.8, 29.4, and 46.9% in LA1, CIN1, and NY1, respectively.

## Discussion

Three different strains of bed bugs with different initial levels of susceptibility to Temprid SC incurred significant life history costs after selection with this pyrethroid/neonicotinoid combination product^22^. The unselected strains varied in their levels of resistance to Temprid SC as is reflected in the exposure times that were required to lead to ca. 80% mortality (i.e., LA1<CIN1<NY1). These initial differences in resistance were inversely related to the initial *R*_*o*_s of these strains (i.e., ET_80_s of 0.1 h, 1 h, and 19 h and *R*_*o*_s of 59.2 (±5.2), 52.6 (±6.4), and 38.7 (±5.6) for LA1, CIN1, and NY1, respectively). Similarly, an earlier study investigating population growth potential of different strains of bed bugs with different insecticide susceptibility profiles found that insecticide resistant strains had fitness costs compared to more susceptible strains^25^. Further, and more convincing, support for the hypothesis of a cost associated with resistance comes from the observation that parental selection for resistance resulted in decreased *R*_*o*_s in the F_2_ of all three populations. Similar tradeoffs between insecticide resistance and life history parameters have been recorded in other insect pests^3^,^4^,^5^,^6^,^7^,^8^,^26^.

In this system, one possible mechanism of the observed costs could be a tradeoff between production of detoxifying enzymes and allocation of resources for fecundity. A previously published study investigating the molecular mechanisms of resistance in the CIN1 selected strain found that four cytochrome P450s and one carboxylesterase was significantly over expressed compared to the CIN1 unselected line^27^. Increased detoxification is likely at least one mechanism of resistance in the LA1 and NY1 selected strains as well. Research investigating mechanisms of resistance in many populations of *C. lectularius* found that the P450 class of enzymes frequently confers a level of resistance to pyrethroid insecticides^16^,^27^,^28^,^29^,^30^,^31^. Given this information, resources may be shunted from fecundity to the production of detoxifying enzymes. This implies a significant metabolic cost associated with the production of these enzymes. Further experiments involving the use of RNA interference could be used to elucidate the molecular mechanism of the observed costs and their correlation with detoxification-mediated insecticide resistance.

The *R*_*o*_ ratios of selected and unselected lines are 0.70, 0.42 and 0.57 for LA1, CIN1 and NY1, respectively. Numbers greater than 1 suggest an advantage, whereas numbers less than one suggest a disadvantage. Thus, a reversion to susceptibility should occur rapidly in insecticide-free environments. We calculated the time course to 50 and 90% recovery of pre-insecticide selection levels of susceptibility using a model proposed earlier^32^.


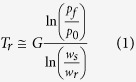



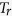
 denotes the time required to reach a significant degree of susceptibility, 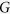 denotes generation time, 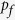 denotes the proportion of the population required to be susceptible, 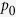 denotes the initial proportion susceptible, 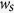 denotes the fitness of the susceptible and 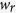 denotes the fitness of the resistant. The *R*_*o*_s for each treatment and strain were used for the fitness variables 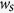 and 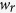. A return to 50% of pre-selection levels of susceptibility would take 3.03, 4.88 and 0.71 generations for LA1, CIN1 and NY1, respectively. Additionally, the same model predicted that the number of generations required for a strain to return to 90% level of pre-selection susceptibility was 6.51, 5.95 and 2.06 for LA1, CIN1 and NY1, respectively. Given that *G*s were less than 20 weeks, reversion to 90% pre-selection levels of resistance should occur within ca. 1 to 2.5 years in the absence of selection.

Currently, the combination pyrethroid/neonicotinoid products are some of the most effective choices for control in the field^21^,^22^. In theory, rotation to products utilizing alternative modes of action could reverse resistance^3^,^10^,^11^ assuming that alleles for susceptibility still exist in the population. However, such reversion is complicated by public intolerance for resident populations (which contrasts with agricultural resistance management^11^), limited gene flow that would help re-establish susceptibility^33^, limited choices of alternative chemical classes^21^ and independence of actions of pest managers when a more coordinated approach might be required. Despite the complications, integration of, or rotation among, both chemical and non-chemical approaches (heat, vacuuming, encasement of beds, etc.) will be necessary for sustained management of populations of bed bugs.

## Material and Methods

### Insects

**Figure 3:**
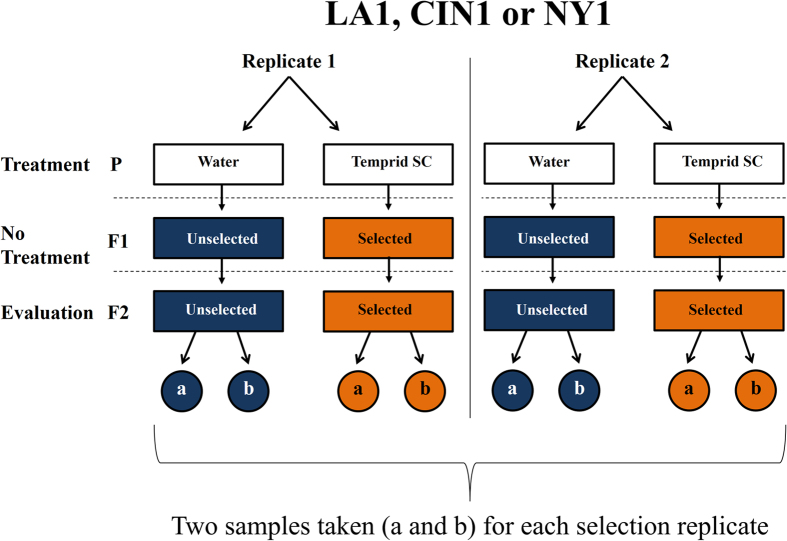
Experimental design for selection experiment. Bugs were exposed to label rate Temprid SC^®^ for a time calculated to kill 80% of the respective strain of bed bugs^22^. Two parental lines of selected and unselected bugs were created for each strain. Using the subsequent F_2_ generation from these lines that receive no further exposure to insecticide, two samples were taken from each replicate to generate data. Replicates were not synchronous and separated by weeks.

Three strains of bed bugs were used for this study. The LA1 strain was collected from Los Angeles in 2007 and was susceptible to pyrethroids^13^. The strain CIN1 was originally collected from Cincinnati, OH in 2005 and was resistant to pyrethroids^13^. Subsequently, its originally high level of pyrethroid resistance has declined but not to the level of the susceptible colony LA1^27^. The NY1 strain was collected from New York City, NY in 2007 and was resistant to pyrethroids^17^. However, a reversion toward susceptibility has also been recorded for this strain, though not to the degree of CIN1^27^. For each strain, two samples of bugs were selected overtime, and two separate lineages of selected and unselected strains were initiated for each strain (LA1, CIN1 and NY1; Fig. 3) by exposing these strains to residual deposits of the pyrethroid/neonicotinoid combination product Temprid SC for a time calculated to kill 80% of the population (ET_80_) at the label rate^22^. All subsequent evaluations of susceptibility were performed using the same exposure times and bioassay. Bugs from an untreated F_1_ generation were used to establish an F_2_ generation (Fig. 3). Insects were housed in incubators away from any insecticide exposure at 26.7° C, 65 ± 5% RH, and a photoperiod of 14:10 (L:D) h. All bed bugs were fed weekly on defibrinated rabbit blood (Quad Five, Ryegate, MT) that was warmed to 39 °C with a circulating water bath using an artificial membrane feeder^34^.

### Life history variables

Life history data were collected for each strain and treatment. A group of 20 eggs was gathered within a 24 hour period from three to 12 females (Supplemental Table S2) and maintained within a small petri dish (5.1 cm diameter) lined with black filter paper. These eggs were allowed to hatch and the resulting individuals were fed weekly for the duration of their lives. Adult offspring were allowed to mate *ad libitum*. Longevity and fecundity were recorded weekly in order to calculate per cent hatching, per cent reaching adulthood, proportion of females, *G*, *R*_*o*_ and oviposition duration. In addition, this information was used to generate weekly survival (*l*_*x*_; female specific survival could not be determined initially due to a lack of information about sex until eclosion to the adult stage) and fecundity (*m*_*x*_; in this case oviposition rates). These recorded values were used to calculate net *R*_*o*_ as the number of female offspring per individual per generation. Because sex determination was only made during the adult stage, estimates of *l*_*x*_, *m*_*x*_, and *R*_*o*_ were based on an assumption of a 1:1 sex ratio of eggs laid by the F_2_ generation. The F_2_ generation for each replicate was followed for up to 70 weeks when the last individual died. Within strains, samples of replicates 1 and 2 were separated in time by 2 to 4 weeks.

### Data analysis

A nested analysis of variance was used to investigate the effects of strain, treatment and replicate(strain) on the hatch rate of eggs laid by F_1_ mothers, the per cent of those eggs that reach adulthood, the observed sex ratios after reaching maturity, *G*, *R*_*o*_ and oviposition duration [Systat software. SYSTAT 13. San Jose, CA (2008)]. Net *R*_*o*_ was calculated as the sum of the weekly *l*_x_*m_*x*_ for each replicate with the assumption of a 1:1 sex ratio of eggs laid by F_2_ females. Generation time was calculated by taking the summation of *l*_x_*m_x_*x divided by the summation of *l*_x_*m_*x*_^35^. A Z-test was performed to investigate the contrast in susceptibility to Temprid SC at the ET_80_ of unselected and selected bugs in the F_2_ generation [Analytical Software. Statistix 8.0 for Windows. Tallahassee, FL (2003)]. Survival analysis was performed to investigate the effects of selection and strain on longevity [JMP^®^ version 11.2.0. SAS Institute Inc., Cary, NC (2013)].

## Additional Information

**How to cite this article**: Gordon, J. R. *et al*. Insecticide resistance in the bed bug comes with a cost. *Sci. Rep*. **5**, 10807; doi: 10.1038/srep10807 (2015).

## Supplementary Material

Supplementary Information

## Figures and Tables

**Table 1:** Percent mortality ± s.e.m. of all strains in the selected and unselected (parental and F_2_) groups and Z-statistics for the F_2_ generation.

Strain^a^	Parental	F2			
		Unselected^b^	Selected	Z_Rep 1_^c^	Z_Rep 2_
LA1	82.2 (±0.4)	83.3 (±6.7)	30.0 (±5.0)	7.20^*^	4.60^*^
CIN1	83.1 (±0.8)	81.7 (±0.0)	3.4 (±3.4)	7.20^*^	8.27^*^
NY1	86.4 (±1.7)	79.2 (±4.2)	36.7 (±8.4)	5.11^*^	4.38^*^

*p < 0.001

^a^Mortality of strains was evaluated following the protocol of Gordon *et al*.^22^ by exposing groups of bugs to residual deposits of Temprid SC. Individuals from each strain were exposed for strain-specific exposure times (LA1 0.1 h, CIN1 1 h, NY1 19 h) calculated to kill 80 per cent of the population.

^b^Average mortality of selection replicates 1 and 2 to strain specific ET_80_s.

^c^A test for a significant difference between selected and unselected proportions in the F_2_ generation for each replicate and strain [Analytical Software. Statistix 8.0 for Windows. Tallahassee, FL (2003)]. N = 60 for each replicate, strain and treatment.
