# Supplementary material for: Insecticide resistance in the bed bug comes with a cost
Source: Sci Rep. 2015 Jun 3;5:10807. doi: 10.1038/srep10807 (PMC4454143; doi:10.1038/srep10807)
Supplement: Supplementary Information [file srep10807-s1.pdf]

Insecticide resistance in the bed bug comes with a cost

Jennifer R. Gordon, Michael F. Potter and Kenneth F. Haynes

Supplemental Table S1. Descriptive statistics for the effects of treatment, strain and replicate(strain) on six different life history parameters

Parameter[a]	Source of variation	Sum of squares	d.f.	Mean square	F-stat	Significance
**% Hatching**						
	Treatment	16.67	1	16.67	0.23	0.636
	Strain	358.33	2	179.17	2.50	0.112
	Replicate(strain)	300.00	3	100.00	1.39	0.279
**% Adult**						
	Treatment	1.04	1	1.04	0.01	0.925
	Strain	758.33	2	379.17	3.30	0.062
	Replicate(strain)	3,284.38	3	1,094.79	9.52	0.001
**% Female**						
	Treatment	0.10	1	0.10	7.00	0.017
	Strain	0.00	2	0.00	0.01	0.982
	Replicate(strain)	0.03	3	0.01	0.74	0.542
**Generation time[b]**						
	Treatment	31.89	1	31.89	7.98	0.012
	Strain	11.38	2	5.69	1.42	0.268
	Replicate(strain)	12.53	3	4.18	1.04	0.398
**Reproductive rate[c]**						
	Treatment	1,362.45	1	1,362.45	12.17	0.003
	Strain	2,078.11	2	1,039.05	9.28	0.002
	Replicate(strain)	821.55	3	273.85	2.45	0.099
**Oviposition duration[d]**						
	Treatment	416.67	1	416.67	9.08	0.008
	Strain	56.583	2	28.29	2.86	0.085
	Replicate(strain)	20.28	3	6.76	1.39	0.279

[a] A nested analysis of variance was used to investigate the effects of strain, treatment and replicate within strain on six life history parameters [Systat software. SYSTAT 13. San Jose, CA

(2008)].

[b] Generation time was calculated by taking the summation of l_x*m_x*x divided by the summation of l_x*m_x[35].

[c] Net reproductive rate (R_o) was calculated as the sum of the weekly l_x*m_x for each replicate with the assumption of a 1:1 sex ratio of eggs laid by F_2 females.

[d] Oviposition duration was calculated by halving the difference of the last week of oviposition by the first week.

Supplemental Table S2. Number of female bed bugs from the F_1 generation used to collect 20 eggs from each replicate and sample

Strain	Treatment[a]	Replicate 1[b]		Replicate 2	
		a[c]	b	a	b
**LA1**					
	Unselected	9	6	12	6
	Selected	6	3	11	6
**CIN1**					
	Unselected	7	9	12	6
	Selected	11	9	11	7
**NY1**					
	Unselected	9	9	9	6
	Selected	9	10	8	7

[a] Selected individuals were exposed to label rate Temprid SC for a time expected to kill 80 % of the respective population[22].

[b] Replicate refers to the asynchronous exposure of two different groups of each strain to Temprid SC.

[c] Refers to the asynchronous collection of 20 eggs from each replicate, treatment and strain.

Supplemental Table S3. Descriptive statistics from survival analysis investigating the effects of strain and selection on longevity

**Strain**[a]	χ^2	**d.f.**	**P-value**
Log-Rank	0.5694	2	0.7522
Wilcoxon	1.1358	2	0.5667
**LA1**[b]	χ^2	**d.f.**	**P-value**
Log-Rank	2.9451	1	0.0861
Wilcoxon	1.1942	1	0.2745
**CIN1**[b]	χ^2	**d.f.**	**P-value**
Log-Rank	0.0061	1	0.9378
Wilcoxon	0.1386	1	0.7097
**NY1**[b]	χ^2	**d.f.**	**P-value**
Log-Rank	0.3067	1	0.5797
Wilcoxon	0.4799	1	0.4884

[a] Survival analysis was performed to investigate differences in longevity between strains [JMP® version 11.2.0. SAS Institute Inc., Cary, NC (2013)].

[b] Survival analysis was performed to investigate the effect of selection on longevity within strain.
